# Molecular epidemiology of MRSA and MSSA in Czech hospitals, 2023: complex genomic epidemiology of CC398 in humans and resurgence of penicillin susceptibility

**DOI:** 10.1093/jac/dkag371

**Published:** 2026-11-13

**Authors:** Adela Rozsypalkova, Pavlina Mrazkova, Julie Cepelikova, Jana Drozenova, Jana Amlerova, Magda Balejova, Ivana Vitkova, Daniela Fackova, Katerina Neradova, Alena Sivakova, Dominika Riedlova, Katerina Fiserova, Natasa Bartonikova, Lenka Dvorakova, Marian Mednansky, Jan Tkadlec, Pavel Drevinek, Oto Melter, Jan Tkadlec

**Affiliations:** Department of Medical Microbiology, Second Faculty of Medicine, Charles University and University Hospital Motol and Homolka, V Uvalu 84, Prague 5, 150 06, Czech Republic; Department of Medical Microbiology, Second Faculty of Medicine, Charles University and University Hospital Motol and Homolka, V Uvalu 84, Prague 5, 150 06, Czech Republic; Department of Medical Microbiology, Second Faculty of Medicine, Charles University and University Hospital Motol and Homolka, V Uvalu 84, Prague 5, 150 06, Czech Republic; Department of Medical Microbiology, University Hospital Bulovka, Prague, Czech Republic; Department of Microbiology, Faculty of Medicine and University Hospital Plzen, Charles University in Prague, Plzen, Czech Republic; Department of Medical Microbiology, Hospital Ceske Budejovice, Ceske Budejovice, Czech Republic; Department of Medical Microbiology, University Hospital Brno, Brno, Czech Republic; Department of Medical Microbiology and Immunology, Hospital Liberec, Liberec, Czech Republic; Department of Clinical Microbiology, University Hospital Hradec Kralove, Hradec Kralove, Czech Republic; Department of Medical Microbiology, Faculty of Medicine, Masaryk University and St. Anne’s University Hospital, Brno, Czech Republic; Department of Microbiology, Agel Laboratories a.s., Ostrava-Vitkovice, Czech Republic; Department of Microbiology, Faculty of Medicine and Dentistry, Palacky University Olomouc, University Hospital Olomouc, Olomouc, Czech Republic; Department of Medical Microbiology, Tomas Bata’s Hospital Zlin, Zlin, Czech Republic; Department of Clinical Microbiology, Masaryk Hospital Usti nad Labem, Usti nad Labem, Czech Republic; Department of Clinical Microbiology, Hospital Havlickuv Brod, Havlickuv Brod, Czech Republic; Department of Clinical Microbiology, Hospital Vsetin, Vsetin, Czech Republic; Department of Medical Microbiology, Second Faculty of Medicine, Charles University and University Hospital Motol and Homolka, V Uvalu 84, Prague 5, 150 06, Czech Republic; Department of Medical Microbiology, Second Faculty of Medicine, Charles University and University Hospital Motol and Homolka, V Uvalu 84, Prague 5, 150 06, Czech Republic; Department of Medical Microbiology, Second Faculty of Medicine, Charles University and University Hospital Motol and Homolka, V Uvalu 84, Prague 5, 150 06, Czech Republic

## Abstract

**Objectives:**

*Staphylococcus aureus* causes a broad spectrum of infections, yet the clonal epidemiology of MSSA remains poorly characterized relative to MRSA. We conducted the first WGS-based national survey of both MRSA and MSSA in the Czech Republic to define contemporary population structure following the COVID-19 pandemic.

**Methods:**

Between September and December 2023, 450 clinical and carriage *S. aureus* isolates were prospectively collected from 14 major hospitals (175 MRSA; 275 MSSA); 248 clinical isolates underwent WGS, with resistance, virulence, mobile genetic elements, SCC*mec* type and clonal complex assignment determined alongside whole-genome MLST and maximum-likelihood phylogeny

**Results:**

MRSA population was less diverse than MSSA (Simpson’s Index of Diversity 0.871 versus 0.986). The historically dominant CC5/ST225-MRSA-II declined sharply, from 80%–90% to 59% of MRSA. CC398 emerged across both phenotypes as three genomically distinct, co-circulating subpopulations: penicillin-susceptible human-adapted MSSA, Panton–Valentine leukocidin (PVL)-positive CA-MRSA (ST1232) and livestock-associated MRSA. PVL-positive MRSA were identified across multiple lineages in 11 hospitals, reflecting a diverse CA-MRSA landscape without dominance of a single clone. Strikingly, nearly half of MSSA isolates (47.6%) were penicillin-susceptible, genotypically confirmed by *blaZ* absence. Penicillin susceptibility was not associated with reduced invasive potential and spanned multiple clonal backgrounds, most prominently CC398 and CC97.

**Conclusions:**

This study reveals a significant shift in *S. aureus* epidemiology: decline of the dominant hospital MRSA clone, emergence of CC398 across the hospital–community–animal interface, and a resurgence of penicillin-susceptible MSSA that challenges the assumption of near-universal penicillin resistance and supports reconsidering penicillin for susceptible infections.

## Introduction

*Staphylococcus aureus* causes infections ranging from mild skin lesions to life-threatening sepsis and pneumonia.^1^ Research has focused preferentially on MRSA, a leading cause of antimicrobial resistance burden.^2^ Yet, while MRSA has declined across Europe for over a decade, MSSA, which causes the majority of invasive infections, is rising,^3^ and MRSA-centric control measures appear to have had little impact on MSSA.^4^ MSSA bacteraemia mortality is comparable to, and in some cohorts exceeds, that of MRSA.^5,6^ Because surveillance centres on bloodstream infections, the epidemiology of the more frequent community-acquired skin and soft tissue infections (SSTIs) remains under-characterized.^7^

Shifts in *S. aureus* epidemiology are typically driven by the emergence, spread or decline of clonal lineages carrying distinct resistance, virulence and mobile genetic element (MGE) repertoires.^8^ CC398 exemplifies this, a heterogeneous complex of sublineages with divergent host preferences and resistance profiles.^9^ It first drew attention in Europe as a livestock-associated lineage (LA-MRSA) among farm workers,^10^ it subsequently gained wider recognition as a cause of human infection through human-adapted MSSA clades,^9^ and most recently, as a Panton–Valentine leukocidin (PVL)-positive hypervirulent sublineage, ST1232-MRSA-V.^11–13^ The regional co-occurrence of these zoonotic and human-adapted subpopulations makes CC398 a critical target for genomic surveillance.

In the Czech Republic, the pre-pandemic MRSA population was dominated by ST225-MRSA-II (CC5),^14,15^ whereas MSSA showed a more heterogeneous structure without a dominant clone.^16^ After the COVID-19 pandemic, invasive MRSA declined nationally and across Europe,^17^ likely reflecting the impact of intensified infection control, the limiting of patient admissions to hospitals and possibly other factors such as the increase in infections caused by Gram-negative bacteria.^17^ At the same time, virulent CA-MRSA sublineages surfaced in the Czech Republic^11^ and Slovakia,^18^ signalling a shift in regional epidemiology.

A less recognized shift in MSSA is the re-emergence of penicillin-susceptible strains (PSSA), with longitudinal increases in penicillin susceptibility^19–21^ reviving narrow-spectrum penicillin as a feasible treatment option.^22^ Whether this is driven by *blaZ* loss or expansion of susceptible lineages remains unknown.

Despite this shifting landscape, a nationally representative, WGS-based characterization of both MRSA and MSSA is lacking for the Czech Republic. Prior work was limited to MRSA, low-resolution typing or pre-pandemic data, leaving the genomic drivers of contemporary trends, such as the emergence of CC398, virulent CA-MRSA and PSSA resurgence, poorly understood. We therefore conducted a multicentre cross-sectional study across 14 major hospitals in 2023 to (i) update the national MRSA population structure after the post-COVID transition; (ii) provide a comprehensive WGS-based characterization of MSSA clonal diversity; and (iii) systematically compare MRSA and MSSA lineages in diversity, resistance and virulence repertoires and MGEs.

## Materials and methods

### Isolate collection

Between September and December 2023, 14 major Czech hospitals (Figure 1) each prospectively collected approximately 10 consecutive single-patient isolates per category: clinical (infection-site) and carriage, MSSA and MRSA, together with anonymized demographic data. Carriage isolates originated from routine diagnostic testing (e.g. admission, pre-operative or in-hospital screening, or incidental isolation from non-infection-associated specimens). Anatomical sites included nasal and pharyngeal swabs (the majority), skin and mucosal swabs (e.g. ear, vaginal) and non-swab specimens (e.g. sputum). Classification as carriage versus infection was made by the submitting clinical microbiologist, based on the absence of clinical signs of infection. Isolates were identified by MALDI-TOF MS. Antimicrobial susceptibility was determined by disk diffusion, including D-tests for inducible clindamycin resistance^23^ and chlorhexidine susceptibility per CLSI.^24^ All 450 isolates underwent PCR for *mecA*, *mecC*, PVL and TSST-1 genes, as described previously.^18^ The clinical isolates were additionally characterized by WGS, from which *spa* type was derived *in silico;*^25^  *spa* typing of the carriage isolates was performed by PCR/Sanger sequencing as described previously.^18^

**Figure 1.**
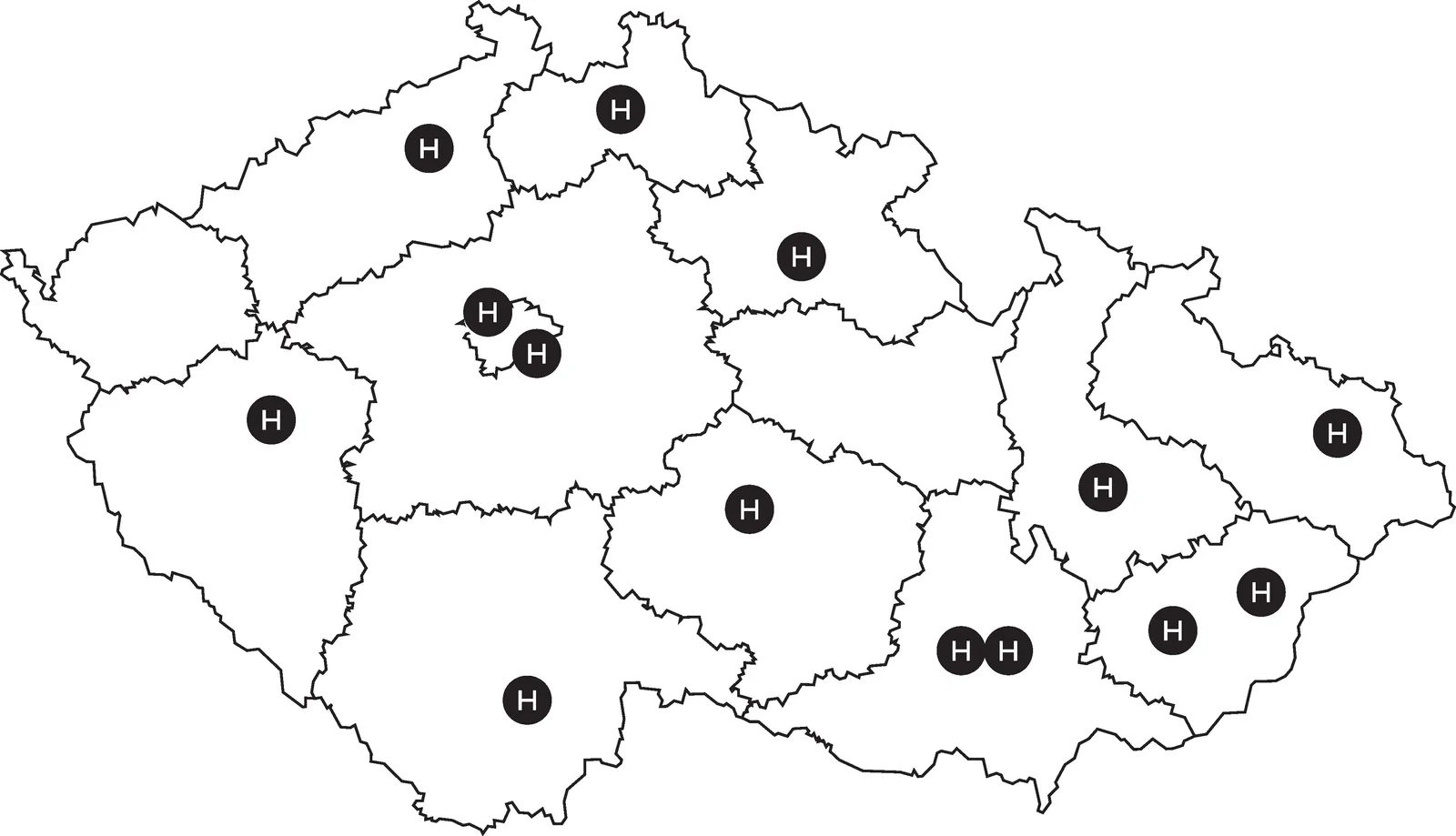
Geographic distribution of the 14 participating hospitals across administrative regions of the Czech Republic. Locations of the 14 hospitals that contributed *Staphylococcus aureus* isolates are shown across 10 of the 14 administrative regions. The base map was obtained from mapsvg.com under the CC0 1.0 Universal licence.

### WGS and analysis

DNA from single colonies (MasterPure Complete DNA and RNA Purification Kit, Biosearch Technologies) was sequenced on an Illumina NextSeq 2000 (DNAprep library kit) and assembled with SPAdes v3.15.5;^26^ assembly quality was checked with QUAST v5.3.0^27^ on Galaxy.^28^ Genomes were screened for virulence^29,30^ and resistance genes,^31^ STs,^32^ MGEs^33^ and SCC*mec* type.^34^ Whole-genome MLST (3897 loci) and minimum spanning trees were generated in BioNumerics v8.1 (bioMérieux), with ≤24 allelic differences indicating close relatedness.^35^ Assemblies were annotated with Bakta v1.9.4;^36^ a core-genome alignment (Roary v3.13.0)^37^ was recombination-filtered with Gubbins v3.2.1;^38^ and a maximum-likelihood phylogeny was inferred and visualized in iTOL v7.2.1.^39^ Heatmaps (Figure 4) and the 2017–18 versus 2023 alluvial comparison (Figure S6, available as Supplementary data at *JAC* Online) were generated in Python 3.12 (matplotlib, seaborn, pandas, numpy). Software versions and settings for all bioinformatic tools are provided in Table S1.

### Statistics

Continuous and categorical variables were compared using Mann–Whitney *U*-test, chi-square or Fisher’s exact test (two-tailed; *P* < 0.05 significant) with Holm–Bonferroni correction, where appropriate. Simpson’s Index of Diversity (SID) with 95% CIs was calculated as described by Grundmann *et al*.^40^ Age-dependent PVL distribution across five age groups was assessed by the Cochran–Armitage trend test.

### Ethics

The project was approved by the Ethics Committee of the Motol University Hospital and Second Faculty of Medicine, Charles University in Prague (approval no. EK—602.35/22).

## Results

### Collection overview and demographics

Of 450 isolates collected (175 MRSA, 275 MSSA), 248 were clinical (104 MRSA, 144 MSSA) and 202 were carriage. Clinical isolates derived mainly from SSTI (66 MRSA/52 MSSA) and bloodstream infections (22/70). Isolates originated from standard wards (*n* = 216), ICU (*n* = 104), ambulatory (*n* = 114) and emergency (*n* = 16) settings. Carriage isolates were significantly over-represented in the ICU compared with the remaining departments [60.6% (63/104) versus 40.2% (139/346); adjusted *P* = 0.004], whereas clinical isolates were over-represented in standard wards [63.4% (137/216) versus 47.4% (111/234); adjusted *P* = 0.004]. The overall male-to-female ratio was 60:40 and median age was 59 years (IQR 30–75) (Table 1). Carriage isolates predominated in children/adolescents (0–17 years) and clinical isolates in seniors (60–74 years), most markedly among MSSA (Table 1).

**Table 1.** Demographic and clinical characteristics of carriage and clinical *Staphylococcus aureus*, Czech Republic, 2023

	MRSA			MSSA			Total (*n* = 450)	*P*-value (MRSA versus MSSA)	*P*-value (Car/Clin)
	Carriage (*n*=71)	Clinical (*n* = 104)	*P*-value (MRSA:Carr./Inv.)	Carriage (*n* = 131)	Clinical (*n* = 144)	*P*-value (MSSA:Carr/Inv)			
Male (%)	41 (57.75)	60 (57.69)	0.994	76 (58.02)	92 (63.89)	0.318	269 (59.78)	0.476	0.468
Female (%)	30 (42.25)	44 (42.31)	0.994	55 (41.98)	52 (36.11)	0.318	181 (40.22)	0.476	0.468
Standard care (%)	38 (53.52)	50 (48.08)	0.479	41 (31.30)	87 (60.42)	**<0.001**	216 (48.00)	1.000	**0**.**004**
Ambulance (%)	12 (16.90)	31 (29.81)	0.160	43 (32.82)	28 (19.44)	**0**.**022**	114 (25.33)	1.000	0.628
ICU (%)	20 (28.17)	16 (15.38)	0.160	43 (32.82)	25 (17.36)	**0**.**009**	104 (23.11)	1.000	**0**.**004**
Emergency (%)	1 (1.41)	7 (6.73)	0.196	4 (3.05)	4 (2.78)	1.000	16 (3.55)	1.000	0.628
Median age (IQR)	60 (19–75)	62 (35–75)	0.535	54 (19–76)	62 (41–73)	0.096	59 (30–75)	0.488	0.085
Children and Adolescents (0–17) (%)	16 (22.54)	12 (11.54)	0.255	27 (20.61)	17 (11.81)	0.188	72 (16.00)	1.000	**0**.**030**
Young adults (18–39) (%)	9 (12.68)	17 (16.35)	1.000	25 (19.08)	16 (11.11)	0.192	67 (14.88)	1.000	0.592
Middle-aged adults (40–59) (%)	10 (14.85)	20 (19.23)	1.000	22 (16.79)	35 (24.31)	0.25	87 (19.33)	1.000	0.270
Seniors (60–74) (%)	18 (25.35)	29 (27.88)	1.000	20 (15.27)	44 (30.56)	**0**.**015**	111 (24.67)	1.000	**0**.**036**
Elderly (75+) (%)	18 (25.35)	26 (25.00)	1.000	36 (27.48)	32 (22.22)	0.313	112 (24.89)	1.000	0.592
PVL (%)	12 (16.90)	30 (28.85)	0.138	5 (3.82)	9 (6.25)	0.606	56 (12.44)	**0**.**002**	**0**.**038**
TSST-1 (%)	5 (7.04)	3 (2.88)	0.272	10 (7.63)	6 (4.17)	0.606	24 (5.33)	0.566	0.075

*P*-values: column ‘*P*-value (MRSA: Carr/Clin)’ tests the difference between MRSA carriage and clinical subgroups (*n* = 71 versus 104); ‘*P*-value (MSSA: Car/Clin)’ tests the equivalent comparison among MSSA isolates (*n* = 131 versus 144); ‘*P*-value (MRSA versus MSSA)’ tests the overall difference between all MRSA (*n* = 175) and all MSSA (*n* = 275); ‘*P*-value (Car/Clin)’ tests carriage versus clinical across the entire cohort (*n* = 202 versus 248). All comparisons used chi-square or Fisher's exact test for categorical variables, and the Mann–Whitney *U*-test for continuous variables; *P*-values were adjusted using Holm–Bonferroni correction; *P*-values < 0.05 are highlighted as statistically significant.

### Phenotypic and molecular characterization of carriage and clinical isolates

#### Phenotypic resistance

MRSA isolates (*n* = 175) were frequently resistant to erythromycin, clindamycin, and ofloxacin, less often to tetracycline and gentamicin, and rarely to other agents (Table S2). MSSA (*n* = 275) were most often resistant to penicillin (*n* = 144), followed by erythromycin, clindamycin, ofloxacin, and gentamicin. Resistance did not differ between carriage and clinical isolates (Table S3). Methicillin resistance was PCR-confirmed in all MRSA: all carried *mecA* except a single *mecC*-positive carriage isolate (t843, CC130).

#### Virulence—PVL and TSST-1

PVL and TSST-1 were detected in 56 (12.4%) and 24 (5.3%) isolates, respectively; both co-occurred in three MRSA (CC22) and one MSSA (CC152). PVL was more prevalent in MRSA (24.0%; *n* = 42) than MSSA (5.1%; *n* = 14; adjusted *P* = 0.002) and in clinical (19.3%; *n* = 39) than carriage (6.9%; *n* = 17) isolates (adjusted *P* = 0.038), and declined sharply with age, from 31.9% (*n* = 23) in children/adolescents to <2% (*n* = 2) in the elderly (Cochran–Armitage; *P* < 0.001). TSST-1, by contrast, was equally distributed across MRSA (4.6%; *n* = 8) and MSSA (5.8%; *n* = 16; adjusted *P* = 0.566) and independent of clinical origin and age (Table 1).

#### Population structure and diversity

Clonal complex (CC) assignment used *spa* types for all 450 isolates, refined by MLST for the 248 WGS isolates (Table S2). MRSA were assigned to 16 CCs, with three lineages (CC5, CC398 and CC59) each >5% of the MRSA, whereas MSSA was more heterogeneous, spanning 24 CCs (six >5%: CC5, CC15, CC30, CC45, CC97 and CC398). While 12 CCs were shared, most dominant lineages showed a clear preference for being MRSA or MSSA, except CC398 and CC8, prominent in both. CC distribution did not differ between clinical and carriage isolates (*P* > 0.05), indicating that the colonizing reservoir mirrors infection-causing strains; CC5 increased with age (Figure 2). Overall *spa* type diversity was high (SID 0.972; Table S4) but diverged sharply by phenotype: MSSA remained near-completely polyclonal (SID 0.986) while MRSA was markedly more clonal (SID 0.871), most so among clinical MRSA (SID 0.832). MSSA diversity was uniformly high across hospitals (SID 0.953–0.996), whereas MRSA diversity varied widely, from 0.993 (hospital 1) to 0.423–0.706 (hospitals 6, 13 and 2), indicating dominant endemic clones in some settings.

**Figure 2.**
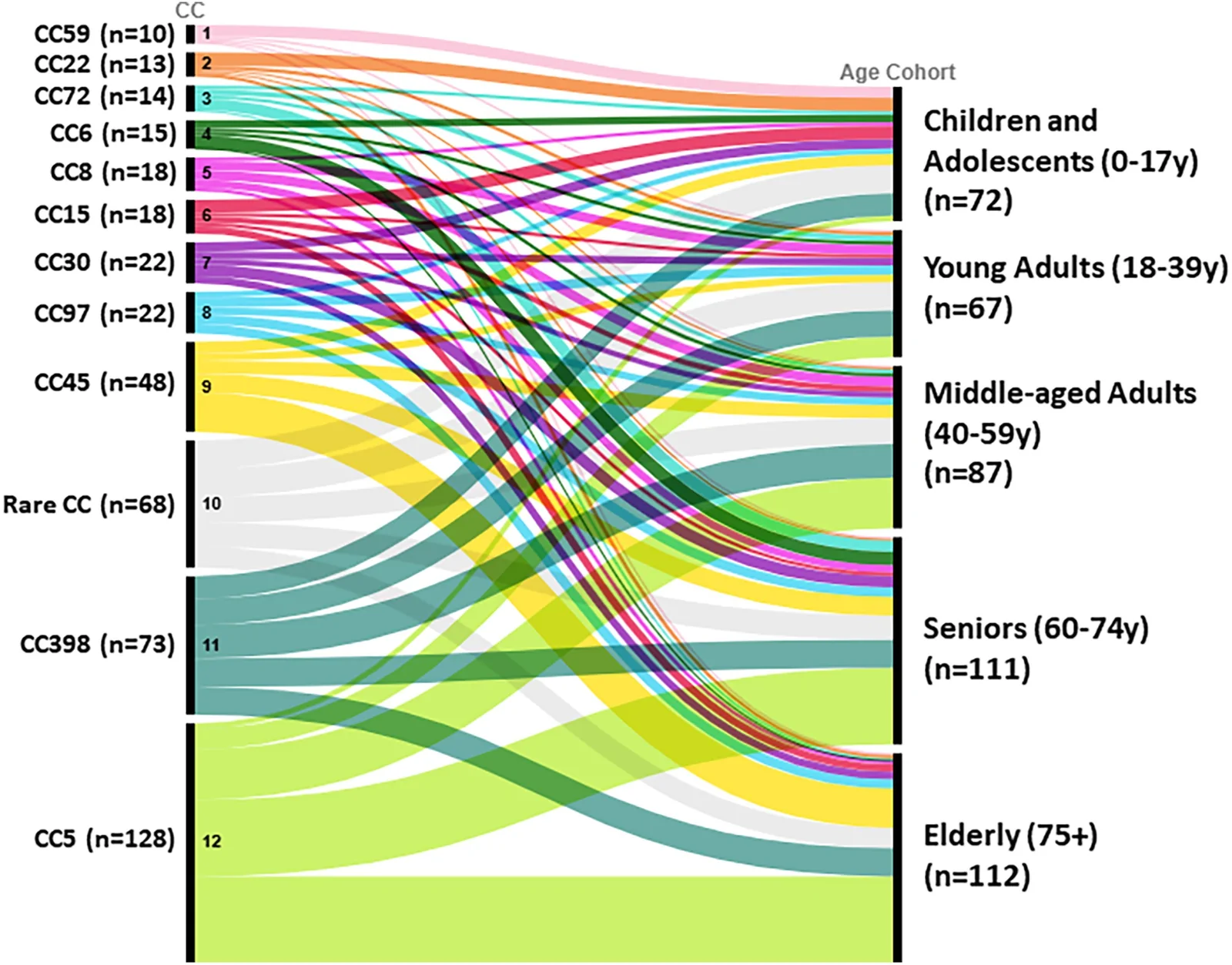
Distribution of major clonal complexes across patient age cohorts. Alluvial diagram showing the relative proportions of the predominant *Staphylococcus aureus* clonal complexes (CCs) among 450 isolates stratified by patient age group. Each stream represents a CC, with stream width proportional to the number of isolates in that cohort. Remaining minor lineages are grouped as ‘rare CCs’.

### Population structure and phylogeny of clinical isolates

Core-genome phylogeny of the 248 clinical isolates (104 MRSA, 144 MSSA) confirmed high clonal diversity (Table S5), with isolates forming well-defined CC-corresponding clusters subdivided by SCC*mec* type, resistance and virulence profiles (Figure 3 and Table 2). Isolates from different hospitals were interspersed without geographic segregation, consistent with inter-hospital circulation of major lineages rather than hospital-specific expansion (Figures S1–S5).

**Figure 3.**
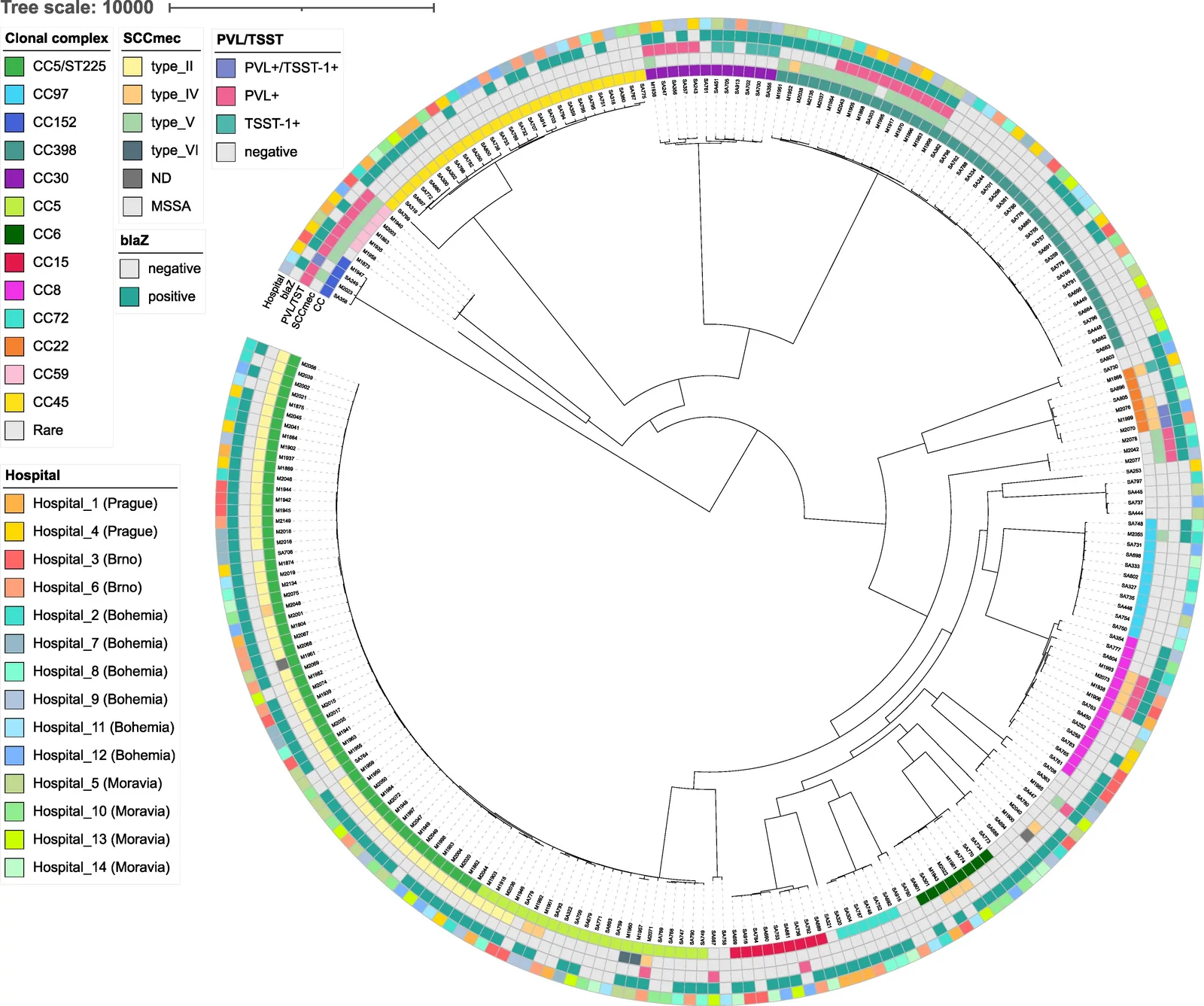
Maximum-likelihood phylogeny of 248 clinical *Staphylococcus aureus* isolates based on a recombination-filtered core-genome alignment, annotated by clonal complex, methicillin susceptibility and key resistance and virulence markers. Genome assemblies were annotated using Bakta, and the core genome alignment was constructed with Roary. Recombinant regions were identified and removed using Gubbins, which simultaneously inferred the maximum-likelihood phylogeny with RAxML under the default settings. The tree was visualized and annotated in iTOL.

**Table 2.** Genomic characterization of major *Staphylococcus aureus* clonal complexes among clinical isolates, Czech Republic, 2023

CC (total *n*)	Lineage type	Median SNP distance (IQR)	Dominant STs (*n*)	SCC*mec* (*n*)	Key resistance genes (*n*)	Key virulence markers (*n*)	MGEs (Tn/IS) (*n*)
CC5 (79)	HA-MRSA (56)	73 (63–85)	ST225 (55)	II (52); - (3); IV(1)	*mecA (53)*; *blaZ (50)*; *erm*(A) (56); *ant*(9)-Ia (56); *gyrA*:S84L (56)	IEC type F (43); *egc (56)*; *sed/sej/ser (44)*; *lukDE (56)*	Tn552 (43); Tn554 (53); Tn6045 (56)
	MRSA/MSSA (23)	237 (219–254)	ST5 (20)	II (4); IV (3); VI (2); - (13)	*mecA (10)*; *blaZ (15)*	IEC variable (B/E/F/G); *egc (23)*; *lukDE (23); lukFS-PV (1)*	Tn6045 (22)
CC398 (42)	CA-MRSA/MSSA (11)	92 (67–101)	ST1232 (11)	V (10); - (1)	*mecA (10)*; *blaZ (11)*; *erm*(A) (11); *ant*(9)-Ia (11); *tet*(K) (8)	IEC type B (11); *lukFS-PV (11)*	Tn554 (7)
	LA-MRSA (5)	115 (59–145)	ST398 (5)	Vc (4); IV (1)	*mecA (5)*; *blaZ (5)*; *lnu*(B) (3); *lsa*(E) (3); *tet*(K) (4); *tet*(M) (5); *dfrG (4)*	IEC negative (5)	Tn552 (4); Tn554 (3); Tn559 (3); Tn6009 (5)
	MSSA (26)	148 (134–172)	ST398 (25)	- (26)	*erm*(T) (21)	IEC type C (23)	—
CC8 (14)	MRSA (4)	121 (83–159)	ST8 (4)	IV (4)	*mecA (4)*; *blaZ (4)*; *msr*(A) (3); *mph*(C) (3); *aac*(6’)-*aph*(2'’) (2); *aph*(3’)-III (3); *gyrA*:S84L (3)	IEC type B (4); ACME (2); *vWbp (4)*; *lukFS-PV (4)*; *lukDE (4)*	Tn552 (3); Tn6216 (3); IS1062 (3)
	MSSA (10)	316 (163–418)	ST8 (7)	- (10)	*blaZ (6)*	IEC type E (7); *lukDE (9)*	Tn6216 (10); IS1062 (8)
CC30 (12)	MSSA/MRSA	449 (231–470)	ST30 (10)	Vc (1); - (11)	*mecA (1); blaZ (10)*	IEC type A (6); *egc (12)*; *vWbp (12)*; *lukFS-PV (5)*; *tst (5)*	Tn552 (6); Tn6216 (11)
CC45 (27)	MSSA	324 (268–364)	ST45 (20)	- (27)	*blaZ (16)*	IEC type B (27); *egc (27)*, *vWbp (26)*	Tn6216 (17)
CC15 (9)	MSSA	264 (120–290)	ST15 (4); ST582 (5)	- (9)	*blaZ (7)*	IEC type C (7); *lukDE (9); lukFS-PV (1)*	Tn6216 (9); IS1062 (9)
CC97 (11)	MSSA/MRSA	108 (68–122)	ST97 (11)	V(1); - (10)	Resistance genes were rare; *mecA* (1)	IEC type E (11); *lukDE (11)*	IS1062 (11)

Genomic features of the seven most prevalent clonal complexes among the 248 clinical *S. aureus* isolates subjected to WGS. CCs comprising distinct subpopulations (CC5, CC398, CC8) are listed separately by methicillin resistance status. Median pairwise SNP distance (IQR) was derived from core-genome SNP analysis. Dominant ST(s) reflect MLST classification, with isolate counts per ST in parentheses. SCC*mec* typing follows IWG-SCC nomenclature; ‘-’ denotes no SCC*mec* detected (MSSA). Resistance and virulence determinants represent markers present in the majority (≥50%) of lineage isolates, except *lukFS-PV*, *tst*, *mecA,* which are reported whenever present regardless of prevalence; ubiquitous (e.g. *hla*) and rare markers are omitted for readability. Mobile genetic elements are summarized in the final column. For a complete overview of detected resistance genes, virulence genes and MGEs, see Table S2.

CA-MRSA, community-associated MRSA; HA-MRSA, healthcare-associated MRSA; IEC, immune evasion cluster (types A–G); IS, insertion sequence; LA-MRSA, livestock-associated MRSA; Tn, transposon.

wgMLST identified thirteen transmission clusters (2–3 isolates each, <24 alleles), spanning ST225-MRSA (*n* = 5), CC5-MRSA/MSSA (*n* = 3), CC398-MSSA (*n* = 2), CC398-CA-MRSA (*n* = 2) and CC45-MSSA (*n* = 1). The predominance of ST225 clusters reflects its known nosocomial propensity, while independent clusters in both CC398 subpopulations indicate ongoing spread of the human-adapted MSSA and CA-MRSA branches (Figures S2–S5). Median within-lineage SNP distances varied markedly (Table 2): CC5/ST225-MRSA was most clonal (73; IQR 63–85), consistent with recent expansion, whereas MSSA-dominated CC30, CC45 and CC8 were far more diverse (449, 324, 316), reflecting long independent histories. CC398 was intermediate, mirroring the distinct origins of its three sublineages, detailed below.

#### MRSA lineages

The clinical MRSA resistome was dominated by genes characteristic of the ST225 clone, including *mecA* (all isolates), *blaZ*, *erm*(A) and *gyrA* p.S84L. Genes *aac*(6′)-*aph*(2″) and *tet*(K) were less frequent; other genes were rare. Chlorhexidine-tolerance *qac* genes occurred in 10 isolates (MIC 2–4 mg/L) (Table S2). CC5 dominated clinical MRSA (60.6%; 63/104) in two sublineages: the large, highly clonal ST225-MRSA-II (*n* = 56, including three MSSA), defined by *mecA*, *blaZ*, *erm*(A), *ant*(9)-*Ia*, Tn554, *gyrA* p.S84L, the *egc* cluster and IEC type F; and a more diverse ST5-MRSA group (*n* = 23; median SNP 237) with variable SCC*mec* (II/IV/VI/none) and IEC types. Beyond CC5, MRSA comprised a diverse, PVL-positive set of CA-MRSA: PVL occurred in 30 clinical MRSA across 10 CCs (CC398 *n* = 10, CC59 *n* = 5, CC8 *n* = 4, CC1153 *n* = 3, CC22 *n* = 2, CC152 *n* = 2 and single CC1/CC121/CC30/CC5). CC59-MRSA (SCC*mec* V) and CC8-MRSA (SCC*mec* IV) were PVL positive with IEC type B, consistent with international CA-MRSA. No single CA-MRSA clone dominated; 10 co-circulating PVL-positive clones spanned 11 hospitals.

#### MSSA lineages

The MSSA resistome was dominated by *blaZ* (*n* = 75) and *erm*(T) (*n* = 21); *qac* genes occurred in four isolates, and others were rare (Figure 4 and Table S2). CC398 was the most prevalent MSSA lineage (27/144; 18.8%) and is detailed below. CC45 (*n* = 27, ST45; median SNP 324) carried a complex enterotoxin repertoire (*egc* with *sec*/*sel* variants), IEC type B, *vWbp* and frequent *aac*(6′)-*aph*(2″) and was largely penicillin-resistant. CC30 (*n* = 12; median SNP 449) was the most toxigenic lineage, isolates frequently carrying *tst* or PVL, and IEC type A. CC15 (*n* = 9; median SNP 264) and CC97 (*n* = 11; median SNP 108) were the most genomically lean: CC15 carried only *blaZ,* IEC type C and *lukDE*, while CC97 had essentially no resistance genes, retaining IEC type E and *lukDE*. The low SNP distance of pan-susceptible CC97 suggests recent clonal expansion rather than resistance loss. CC8-MSSA (*n* = 10; median SNP 316) was the most diverse and clustered with CC8-MRSA, indicating co-circulation within CC8. Overall, MSSA lineages spanned divergent strategies, from the toxigenic CC30 to the lean, pan-susceptible CC97.

**Figure 4.**
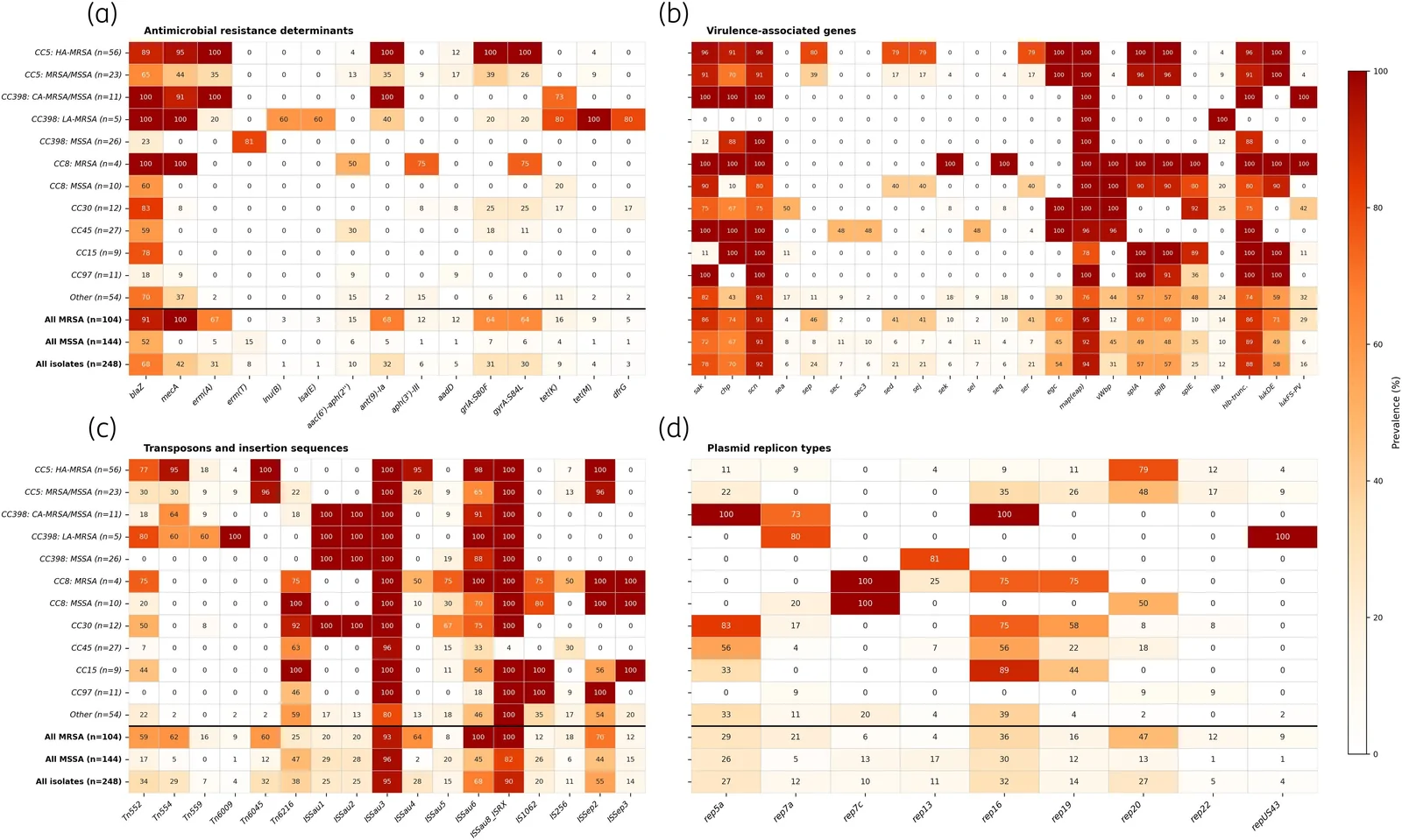
Lineage-stratified distribution of antimicrobial resistance determinants, virulence genes, mobile genetic elements and plasmid replicon types among 248 clinical *Staphylococcus aureus* isolates. Heatmaps display the prevalence (%) of selected markers across major clonal complexes and methicillin susceptibility categories. (a) Antimicrobial resistance genes and resistance-associated point mutations. (b) Virulence-associated genes. (c) Transposons and insertion sequences. (d) Plasmid replicon types. Colour intensity reflects prevalence within each lineage (0%–100%, shared colour scale). Genes ubiquitously present (≥95% of all isolates, Table S2) are not shown. Markers were included if detected in ≥5% of all isolates or if exhibiting lineage-defining distribution (≥50% prevalence within a lineage of *n* ≥ 5). Summary rows show aggregate prevalence for all MRSA (*n* = 104) and all MSSA (*n* = 144) isolates. Three phenotypically MSSA isolates (M1961, SA706, SA764) clustering within CC5 HA-MRSA are tabulated under that lineage.

#### CC398—a lineage bridging MRSA and MSSA

Among 42 clinical CC398 isolates, three genomically distinct subpopulations were identified, two sharing ST398 but differing in accessory genome and the distinct ST1232 sublineage (Table 2 and Figure 4). Human-adapted CC398-MSSA (*n* = 26, ST398/ST1570; median SNP 148) was the largest, defined by IEC type C and *erm*(T) as the only common resistance gene, contributing substantially to the penicillin-susceptible reservoir. CC398-CA-MRSA (*n* = 11, ST1232; median SNP 92) carried SCC*mec* V and uniformly PVL and IEC type B, with a consistent *blaZ*, *erm*(A), *ant*(9)-*Ia*, *tet*(K), Tn554 profile; one phenotypically MSSA ST1232 isolate clustered here, suggesting SCC*mec* loss or an MSSA ancestor. Its low diversity is consistent with recent clonal expansion of this hypervirulent sublineage. CC398-LA-MRSA (*n* = 5, ST398; median SNP 115) carried SCC*mec* Vc/IV and the classical livestock profile—IEC negative, *tet*(M)/*tet*(K) positive with an extended resistance and transposon repertoire (*lnu*(B), *lsa*(E), *dfrG*; Tn552, Tn554, Tn559, Tn6009); three of five came from a single North-East Bohemia hospital, suggesting a regional reservoir. wgMLST found transmission clusters in the MSSA (*n* = 2) and CA-MRSA (*n* = 2) subpopulations but none among LA-MRSA.

#### Re-emerging PSSA

Approximately half of MSSA isolates (131/275; 47.6%) were phenotypically penicillin-susceptible, genotypically confirmed by *blaZ* absence in 69/144 sequenced isolates (47.9%). PSSA spanned multiple clonal backgrounds, with dominant lineages containing both *blaZ*-positive and *blaZ*-negative isolates (Figure 3), but was significantly enriched for CC398 (30.5% versus 8.3%; *P* < 0.001) and CC97 (12.2% versus 3.5%; *P* = 0.013), whereas PR-MSSA was associated with CC30 (*P* = 0.005) (Table 3). *tst* (*P* = 0.002) and the *egc* cluster (*P* = 0.004) were enriched in PR-MSSA, IEC type E in PSSA (*P* = 0.002) and type B in PR-MSSA (*P* = 0.004). However, sex, age, proportion of clinical isolates (52.7% versus 52.1%) and type of care did not differ.

**Table 3.** Demographic, clinical, clonal and genomic characteristics of penicillin-susceptible *Staphylococcus aureus* (PSSA) compared with penicillin-resistant (PR-)MSSA (*blaZ*-positive) isolates, Czech Republic, 2023

		PSSA (*n* = 131)	PR-MSSA (*n* = 144)	*P*-value
Sex	Male	84 (64.12)	84 (58.33)	0.325
	Female	47 (35.88)	60 (41.67)	0.325
Type of care	Standard care	68 (51.91)	60 (41.67)	0.356
	Ambulance	28 (21.37)	43 (29.86)	0.356
	ICU	30 (22.90)	38 (26.39)	0.968
	Emergency	5 (3.82)	3 (2.08)	0.968
Age	Median age (IQR)	55 (30–71)	60 (26–75)	1.000
	Children and adolescents (0–17)	19 (14.50)	25 (17.36)	1.000
	Young adults (18–39)	20 (15.27)	21 (14.58)	1.000
	Middle-aged adults (40–59)	32 (24.43)	25 (17.36)	0.894
	Seniors (60–74)	32 (24.43)	32 (22.22)	1.000
	Elderly (75+)	28 (21.37)	40 (27.78)	1.000
Clonal background	CC15	5 (3.82)	13 (9.03)	0.243
	CC30	2 (1.53)	17 (11.81)	**0.005**
	CC398	40 (30.53)	12 (8.33)	**<0.001**
	CC45	15 (11.45)	32 (22.22)	0.071
	CC5	13 (9.92)	11 (7.64)	0.994
	CC8	5 (3.82)	8 (5.56)	0.994
	CC97	16 (12.21)	5 (3.47)	**0**.**013**
Virulence	PVL	6 (4.58)	8 (5.56)	1.000
	TSST-1	1 (0.76)	15 (10.42)	**0**.**002**
	Clinical isolates	69 (52.67)	75 (52.08)	1.000
				
Sequenced isolates		*blaZ*-(*n* = 69)	*blaZ*+ (*n* = 75)	
Virulence genes	IEC type B	17 (24.64)	36 (48.00)	**0**.**004**
	IEC type E	21 (30.43)	6 (8.00)	**0**.**002**
	Egc	22 (31.88)	43 (57.33)	**0**.**004**

All comparisons used chi-square or Fisher's exact test for categorical variables, and the Mann–Whitney *U*-test for continuous variables; *P*-values were adjusted using Holm–Bonferroni correction; *P*-values <0.05 are highlighted as statistically significant.

*blaZ*, β-lactamase gene; *egc*, enterotoxin gene cluster; IEC, immune evasion cluster (types A–G); PR-MSSA, penicillin-resistant MSSA; PSSA, penicillin-susceptible *S. aureus*; PVL, Panton–Valentine leukocidin; TSST-1, toxic shock syndrome toxin-1.

#### Lineage-dependent distribution of MGEs, virulence factors and resistance determinants

The resistome, virulome and mobilome followed clonal patterns (Figure 4 and Tables S6–S9). Plasmid replicons were lineage-specific (rep20 in CC5 HA-MRSA, rep7c universal in CC8, rep16 in CC15, rep13 with *erm*(T) in CC398 MSSA, repUS43 marking CC398 LA-MRSA). Tn554 marked CC5 HA-MRSA (with erm(A)/ant(9)-Ia), while ISSau1 and ISSau2 were exclusive to CC398 and Tn559 and Tn6009 characterized CC398 LA-MRSA; CC97, CC15 and CC45 MSSA carried minimal accessory resistance. IEC genes were present in 92.7% of isolates, predominantly type C, with type F strongly associated with CC5 HA-MRSA. egc was nearly universal in CC5, CC30 and CC45 (≥99%) but absent in other main lineages, while splE was enriched in MSSA backgrounds (CC30, CC15 and CC8).

## Discussion

CC5, predominantly ST225-MRSA-II, remained dominant MRSA but has declined substantially from 81.4% (359/441) in our 2017–18 national survey^14^ (Figure S6) and up to 91.4% (565/618) in a contemporaneous bloodstream survey^15^ to 59.4% (104/175) in 2023. WGS resolved CC5 into distinct subpopulations: the dominant ST225-MRSA-II group (including three phenotypically MSSA isolates) and a more diverse ST5 MRSA/MSSA cluster. Despite its strong association with the MRSA phenotype (*P* < 0.001), CC5 was the third most prevalent lineage among MSSA overall, after CC398 and CC45. The decline of CC5/ST225 mirrors the broader MRSA decline across Europe,^17^ likely facilitated by pandemic-era infection control.^41^ Crucially, ST225 has not been replaced by a single successor; instead, the MRSA population has diversified, the largest gain being CC398, alongside smaller expansions of CC6, CC22 and CC59 (Figure S6).

CC398 emerged as three distinct sublineages. Typical LA-MRSA (*tet*(M), SCC*mec* IV/Vc, IEC negative)^9^ was rare (five isolates); three from a single North-East Bohemia hospital without evidence of transmission, as CC398 was already over-represented in this region previously,^14^ representing a sustained reservoir linked to local livestock. Consistently with recent reports,^11,13^ we confirmed the presence of ST1232, a PVL-positive human-adapted CC398 sublineage common in Southeast Asia^42^ across five hospitals (*n* = 11), predominantly in skin and soft-tissue infections, indicating wider geographic spread. The largest subpopulation was the human-adapted CC398 MSSA, characterized by *erm*(T), otherwise rare in *S. aureus,*^16^ and absence of *blaZ*.

Among the remaining MRSA, most belonged to CA-MRSA lineages. Whereas CA-MRSA epidemiology was previously characterized by dominance of a single successful clone, such as ST80 in Greece,^43^ USA300 (ST8) in North America^44^ and Slovakia,^18^ we found multiple co-circulating lineages without clear dominance. Several (CC59, CC8/USA300-like, CC30) were already present in our 2017–18 study,^14^ suggesting a stable reservoir.

Beyond Asia-linked ST1232 and CC59,^42,45^ three further lineages of foreign origin point to global CA-MRSA circulation. ST22-MRSA-IV (predominantly t223) was more common than previously (Figure S6). Several observations are consistent with, though do not prove, importation linked to Ukrainian war refugees: a post-2022 rise in the previously rare t223, its reported frequency as a CA-MRSA in western Russia;^46^ and ST22 MRSA in Ukrainian refugees hospitalized in the Netherlands.^47^ However, variable PVL and TSST-1 carriage suggest several emerging ST22 subclones.^48–50^ CC1153-MRSA-V (*n* = 4, t903, PVL positive), a Middle East/Arabian Gulf lineage only sporadically reported in Western Europe,^51^ was detected for the first time in four unrelated patients across several hospitals, suggesting independent importations. Finally, CC6 (mainly t304), near-absent in 2017–18 but expanding in 2023, has been linked to the Middle East despite rising across Northern Europe.^52^ Three unrelated lineages with distinct geographic origins support international travel and migration as an important driver of the CA-MRSA epidemiology.

Systematic inclusion of MSSA revealed a strikingly polyclonal population (SID 0.986) with no dominant lineage, consistent with other European surveys.^53^ Most lineages exhibited substantial within-lineage diversity (median SNP distances 316–449 in CC8, CC30 and CC45), indicating long-term human circulation. CC45 is a major and persistent MSSA lineage in northern and central Europe.^53^ The exception was CC97 with low within-lineage diversity (108 SNPs) and near-absent resistance and virulence genes, indicating recent clonal expansion. Historically linked to bovine mastitis and human–animal transmission,^54^ the expansion of pan-susceptible CC97 alongside CC398 MSSA points to a population-level shift towards penicillin-susceptible *S. aureus*.

Nearly half of MSSA isolates (47.6%) were penicillin-susceptible, consistent with *blaZ* detection in only about half of sequenced MSSA, ruling out cryptic *blaZ* missed by phenotypic testing.^55^ Historically, penicillin resistance was so prevalent that many laboratories stopped routine testing. Fewer than 10% of US isolates were susceptible in the late 1980s,^56^ and the global TEST survey reported 9.8% with only a slight rise to 2011.^57^ Detected PSSA prevalence is markedly higher than most recent reports, though the upward trend agrees with data from Australia,^21^ the USA,^19^ China^20^ and Spain,^58^ and supports re-evaluating penicillin for susceptible *S. aureus*, reinforced by recent clinical trials.^22,59^ Unlike reports linking PSSA to increased infection severity,^60^ we found no difference in the proportion of penicillin-susceptible versus resistant isolates among clinical infections; instead, *tst* and *egc* were enriched in penicillin-resistant MSSA. Similarly, in contrast to reports of clonal expansion within CC5 and CC45,^61^ the rise in penicillin susceptibility in our cohort reflected a broad epidemiological trend across diverse lineages rather than expansion of a single clone.

Our study has several limitations. Participating hospitals differ in size and catchment, which may influence overall MRSA/MSSA prevalence. The four-month collection window may have missed seasonal variation. WGS was restricted to the 248 clinical isolates, potentially under-representing carriage-only lineages. Finally, limited clinical metadata (e.g. travel history) means the proposed links between specific clones (CC22, CC1153, CC6) and migration remain unconfirmed.

### Conclusions

This first national WGS survey of both MRSA and MSSA in the Czech Republic documents fundamental shifts in the *S. aureus* population: a decline of the dominant ST225-MRSA-II clone, the rise of three distinct CC398 subpopulations, and the emergence of various CA-MRSA (ST1232, CC59, CC22, CC1153, CC6). The parallel resurgence of penicillin susceptibility supports reassessing penicillin clinically, and together these findings argue for an MSSA-inclusive approach to *S. aureus* genomic surveillance.

## Supplementary Material

dkag371_Supplementary_Data

## Data access

Raw sequence reads and genome assemblies of all isolates were submitted to the NCBI Sequence Read Archive and GenBank, respectively, under BioProject accession number PRJNA1472394.

## Supplementary data

Figures S1–S6 and Tables S1–S5 are available as Supplementary data at *JAC* Online.
