# Supplementary material for: Molecular epidemiology of MRSA and MSSA in Czech hospitals, 2023: complex genomic epidemiology of CC398 in humans and resurgence of penicillin susceptibility
Source: J Antimicrob Chemother. 2026 Nov 13;81(12):dkag371. doi: 10.1093/jac/dkag371 (PMC13759972; doi:10.1093/jac/dkag371)
Supplement: Supplementary file 1 [file dkag371_supplementary_data.zip › Supplementary_figures.docx]

Supplementary figure S1. Minimum spanning tree of 248 clinical *S. aureus* isolates based on whole-genome MLST

Allelic profiles were determined and the minimum spanning tree was constructed using Bionumerics v8.1 (bioMérieux, Applied Maths). Each node represents a single isolate; node colour indicates the contributing hospital. Branch lengths correspond to the number of allelic differences between isolates. Clusters of closely related isolates (≤24 allelic differences) are highlighted by shaded backgrounds, indicating potential epidemiological links.

**Alt-text:** Minimum spanning tree of 248 clinical *S. aureus* isolates based on whole-genome MLST, with nodes coloured by contributing hospital and clusters of closely related isolates highlighted.

Supplementary figure S2. Minimum spanning tree of 79 clinical *S. aureus* Clonal complex 5 isolates based on whole-genome MLST

Allelic profiles of all CC5 isolates (56 HA-MRSA and 23 MRSA/MSSA) were determined using the S. aureus wgMLST, the minimum spanning tree was constructed using Bionumerics v8.1 (bioMérieux, Applied Maths). Each node represents a single isolate; node colour indicates the contributing hospital. Branch lengths correspond to the number of allelic differences between isolates. Clusters of closely related isolates (≤24 allelic differences) are highlighted by shaded backgrounds, indicating potential epidemiological links.

**Alt-text:** Minimum spanning tree of 79 clonal complex 5 isolates based on whole-genome MLST, with nodes coloured by contributing hospital and clusters of closely related isolates highlighted.

Supplementary figure S3. Minimum spanning tree of 42 clinical *S. aureus* Clonal complex 398 isolates based on whole-genome MLST

Allelic profiles of all CC398 isolates were determined using the *S. aureus* wgMLST, the minimum spanning tree was constructed using Bionumerics v8.1 (bioMérieux, Applied Maths). The dataset comprises three epidemiologically distinct sublineages: CA-MRSA/MSSA (n = 11), LA-MRSA (n = 5), and MSSA (n = 26). Each node represents a single isolate; node colour indicates the contributing hospital. Branch lengths correspond to the number of allelic differences between isolates. Clusters of closely related isolates (≤24 allelic differences) are highlighted by shaded backgrounds.

**Alt-text:** Minimum spanning tree of 42 clonal complex 398 isolates based on whole-genome MLST, showing three sublineages (CA-MRSA/MSSA, LA-MRSA, MSSA), with nodes coloured by contributing hospital.

Supplementary figure S4. Minimum spanning tree of 27 clinical *S. aureus* Clonal complex 45 isolates based on whole-genome MLST

Allelic profiles of all CC45 isolates (exclusively MSSA) were determined using the *S. aureus* wgMLST, the minimum spanning tree was constructed using Bionumerics v8.1 (bioMérieux, Applied Maths). Each node represents a single isolate; node colour indicates the contributing hospital. Branch lengths correspond to the number of allelic differences between isolates. Clusters of closely related isolates (≤24 allelic differences) are highlighted by shaded backgrounds.

**Alt-text:** Minimum spanning tree of 27 clonal complex 45 isolates (all MSSA) based on whole-genome MLST, with nodes coloured by contributing hospital.

Supplementary figure S5. Minimum spanning tree of clinical *S. aureus* Clonal complex 8, 15, 30, and 97 isolates based on whole-genome MLST

Allelic profiles were determined using the *S. aureus* wgMLST, the minimum spanning tree was constructed using Bionumerics v8.1 (bioMérieux, Applied Maths). (A) CC8 (n = 14; 4 MRSA, 10 MSSA). (B) CC30 (n = 12; 1 MRSA, 11 MSSA). (C) CC15 (n = 9; all MSSA). (D) CC97 (n = 11; 1 MRSA, 10 MSSA). Each node represents a single isolate; node colour indicates the contributing hospital. Branch lengths correspond to the number of allelic differences between isolates. Clusters of closely related isolates (≤24 allelic differences) are highlighted by shaded backgrounds.

**Alt-text:** Four minimum spanning trees based on whole-genome MLST for clonal complexes 8, 30, 15, and 97, with nodes coloured by contributing hospital.

Supplementary figure S6. Shift in MRSA clonal complex distribution between 2017-2018 and 2023

Alluvial diagram comparing the relative proportions of major clonal complexes among clinical *S. aureus* isolates collected in 2017/2018 (n = 441; Tkadlec et al., 2021) and 2023 (n = 175; present study). Each stream connects the same CC across the two study periods; stream width is proportional to the number of isolates. Minor lineages are grouped as "Other."

**Alt-text:** Alluvial diagram comparing the relative proportions of major MRSA clonal complexes between 2017–2018 and 2023, with stream width proportional to isolate numbers.
